# Breaking of Thermopower–Conductivity Trade‐Off in LaTiO_3_ Film around Mott Insulator to Metal Transition

**DOI:** 10.1002/advs.202102097

**Published:** 2021-10-21

**Authors:** Takayoshi Katase, Xinyi He, Terumasa Tadano, Jan M. Tomczak, Takaki Onozato, Keisuke Ide, Bin Feng, Tetsuya Tohei, Hidenori Hiramatsu, Hiromichi Ohta, Yuichi Ikuhara, Hideo Hosono, Toshio Kamiya

**Affiliations:** ^1^ Laboratory for Materials and Structures Tokyo Institute of Technology 4259 Nagatsuta, Midori‐ku Yokohama 226‐8503 Japan; ^2^ PRESTO Japan Science and Technology Agency 7 Gobancho Chiyoda‐ku Tokyo 102‐0076 Japan; ^3^ Research Center for Magnetic and Spintronic Materials National Institute for Materials Science 1‐2‐1 Sengen Tsukuba Ibaraki 305‐0047 Japan; ^4^ Institute of Solid State Physics Vienna University of Technology Wiedner Hautptstrasse 8‐10, A‐1040 Vienna Austria; ^5^ Graduate School of Information Science and Technology Hokkaido University N14W9, Kita‐ku Sapporo 060‐0814 Japan; ^6^ Institute of Engineering Innovation The University of Tokyo 2‐11‐16 Yayoi, Bunkyo‐ku Tokyo 113‐8656 Japan; ^7^ Graduate School of Engineering Science Osaka University 1‐3 Machikaneyama‐cho Toyonaka Osaka 560‐8531 Japan; ^8^ Materials Research Center for Element Strategy Tokyo Institute of Technology 4259 Nagatsuta, Midori‐ku Yokohama 226‐8503 Japan; ^9^ Research Institute for Electronic Science Hokkaido University N20W10, Kita‐ku Sapporo 001‐0020 Japan

**Keywords:** epitaxial strains, metal–insulator transition, strongly correlated oxide, thermoelectrics, transition metal oxide

## Abstract

Introducing artificial strain in epitaxial thin films is an effective strategy to alter electronic structures of transition metal oxides (TMOs) and to induce novel phenomena and functionalities not realized in bulk crystals. This study reports a breaking of the conventional trade‐off relation in thermopower (*S*)–conductivity (*σ*) and demonstrates a 2 orders of magnitude enhancement of power factor (PF) in compressively strained LaTiO_3_ (LTO) films. By varying substrates and reducing film thickness down to 4 nm, the out‐of‐plane to the in‐plane lattice parameter ratio is controlled from 0.992 (tensile strain) to 1.034 (compressive strain). This tuning induces the electronic structure change from a Mott insulator to a metal and leads to a 10^3^‐fold increase in *σ* up to 2920 S cm^−1^. Concomitantly, the sign of *S* inverts from positive to negative, and both *σ* and *S* increase and break the trade‐off relation between them in the n‐type region. As a result, the PF (=*S* ^2^ *σ*) is significantly enhanced to 300 µW m^−^^1^K^−2^, which is 10^2^ times larger than that of bulk LTO. Present results propose epitaxial strain as a means to finely tune strongly correlated TMOs close to their Mott transition, and thus to harness the hidden large thermoelectric PF.

## Graphical abstract

Thermoelectric power‐factor enhancement by 2 orders of magnitude is discovered beyond the Mott insulator to metal transition in compressively strained LaTiO_3_ films. Both conductivity and thermopower increase in the n‐type region, breaking the conventional trade‐off between them. Tuning oxides close to their Mott transition through epitaxial strain is proposed for harnessing large power factors.

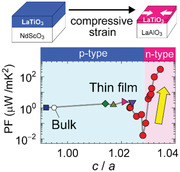

## 1 Introduction

Since the discovery of large thermopower (*S*) accompanied by high electrical conductivities (*σ*) in strongly correlated transition metal oxides (TMOs), such as Na*_x_* CoO_2_,^[^ ^1^ ^]^ the interplay of thermoelectricity and electronic correlations has been recognized as a potential source for high‐performance thermoelectrics.^[^ ^2^, ^3^, ^4^, ^5^ ^]^ Indeed, the coexistence of large *S* and high *σ* causes a large power factor (PF = *S* ^2^⋅*σ*).^[^ ^6^ ^]^ Therefore, strongly correlated TMOs continue to be explored for their thermoelectric properties.

An established way to control the thermoelectric properties of TMOs is carrier doping through the addition of impurity elements, which has led to high PFs in La‐doped SrTiO_3_,^[^ ^7^ ^]^ rare‐earth‐doped CaMnO_3_,^[^ ^8^ ^]^ and Sr‐doped LaCoO_3_.^[^ ^9^ ^]^ However, the enhancement of PF is restricted by the well‐known trade‐off relationship between *σ* and *S*.^[^ ^6^, ^10^ ^]^ Both *σ* and *S* depend on carrier concentration *n* but possess opposite relations based on the carrier‐diffusion model; *σ* increases with growing *n* whereas *S* decreases, limiting the maximum PF at a certain *n*. Thus we can expect decoupling this trade‐off beyond the carrier‐diffusion model will further improve thermoelectric performance of TMOs.

Here, we discovered an unusually large PF enhancement by breaking the trade‐off relation between *σ* and *S* in a lattice‐strain controlled TMO of LaTiO_3_ (LTO). Introducing artificial strain in epitaxial thin film has been an effective strategy to alter electronic structures of TMOs and has shown to manipulate various physical properties of TMOs, such as metal–insulator transitions,^[^ ^11^, ^12^ ^]^ superconducting properties,^[^ ^13^, ^14^ ^]^ and magnetic properties.^[^ ^15^, ^16^, ^17^ ^]^ The effect of epitaxial strain on thermoelectricity, however, is still largely unexplored. The perovskite‐type oxide LTO is a good platform to attempt controlling PF by epitaxial strain. With its Ti 3d^1^ electronic configuration, LTO is a prototypical Mott–Hubbard insulator that orders antiferromagnetically below 146 K.^[^ ^18^, ^19^, ^20^ ^]^ However, its gap of 0.1−0.2 eV^[^ ^21^ ^]^ is uncharacteristically small, hinting at a large tunability of properties by external stimuli. Indeed, LTO's electronic structure sensitively depends on the rotation and tilting angles of the TiO_6_ octahedra in the distorted orthorhombic lattice (space group *Pbnm*).^[^ ^22^ ^]^ Deforming this lattice by external strain hence promises to notably manipulate the thermoelectric response.

Theoretically, it is predicted that an in‐plane compressive strain of about −2% induces a Mott insulator to metal transition in LTO, while tensile strain stabilizes the Mott insulating state.^[^ ^23^ ^]^ Notably large PFs have been suggested to occur near Mott‐insulator to metal transitions in TMOs.^[^ ^24^, ^25^, ^26^ ^]^ On the Mott insulator side, localized electrons may result in large *S* due to sharp density‐of‐states (DOS) features near the Fermi level (*E*_F_), while the metal side with itinerant electrons has large *σ*. Our work is hence motivated by the expectation of an enhanced PF for compressively strained LTO thin films in the proximity of the Mott insulator to metal transition.

## 2 Results and Discussion

**Figure 1:**
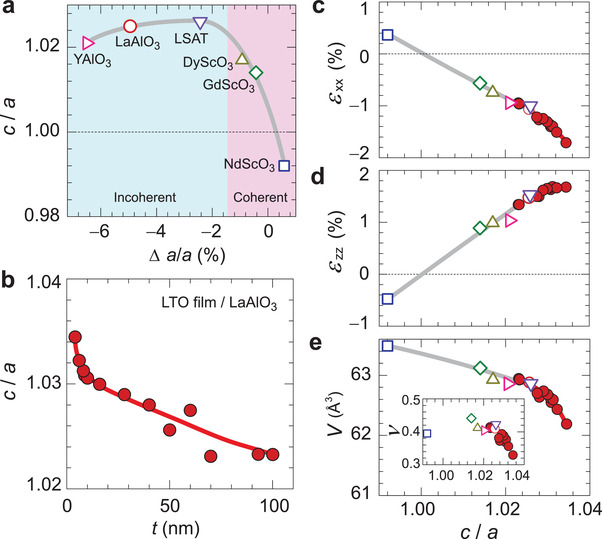
Structural characterization of LTO epitaxial films. a) In‐plane lattice mismatch (Δ*a*/*a*) dependence of the out‐of‐plane to the in‐plane lattice parameter ratio (*c*/*a*) for 50 nm thick LTO films on YAlO_3_, LaAlO_3_, LSAT, DyScO_3_, GdScO_3_, and NdScO_3_ substrates. Red (blue) areas indicate regions with ǀΔ*a*/*a*ǀ < 1 (ǀΔ*a*/*a*ǀ > 1) where epitaxial growth is coherent (relaxed). b) Film thickness (*t*) dependence of *c*/*a* for LTO films grown on LaAlO_3_. c‐e) *c*/*a* dependence of c) in‐plane epitaxial strain (*ε_xx_*), d) out‐of‐plane epitaxial strain (*ε_zz_*), e) lattice volume (*V*) of the pseudo‐cubic lattice. Epitaxial strains *ε_xx_* = (*a*_film_ − *a_c_*_/_ *_a_*_=1_) / *a_c_*_/_ *_a_*_=1_ and *ε_zz_* = (*c*_film_ − *c_c_*_/_ *_a_*_=1_)/*c*_c/a=1_ for in‐ and out‐of‐plane strains, respectively, are evaluated with respect to the pseudo‐cubic (*c/a* = 1) lattice parameters. The inset in (e) shows Poisson's ratio, *ν* = *ε_zz_* / (*ε_zz_* – 2*ε_xx_*).

To control strain, we grew a series of LTO epitaxial films on different (001) pseudo‐perovskite substrates by pulsed laser deposition. To quantify the lattice mismatch, we use the pseudo‐cubic reference, in which the orthorhombic lattice parameters of bulk LTO (*a* = 5.634 Å, *b* = 5.616 Å, *c* = 7.915 Å),^[^ ^27^ ^]^ translate to *a*_bulk_ = $a_{\mathrm{bulk}}=\sqrt{(a^{2}+b^{2})/2}=3.977$ Å and *c*_bulk_ = *c*/2 = 3.958 Å. The in‐plane lattice mismatches, Δ*a*/*a* (=(*a*_sub._ − *a*_bulk_)/*a*_bulk_) for the different substrates are −6.50% (YAlO_3_), −4.95% (LaAlO_3_), −2.44% ((La,Sr)(Al,Ta)O_3_ = LSAT), −0.93% (DyScO_3_), −0.43% (GdScO_3_), and +0.58% (NdScO_3_). For each substrate, the epitaxial growth of 50 nm thick LTO films was confirmed by X‐ray diffraction (Figures S1 and S2, Supporting Information). The in‐plane lattices of the LTO films are coherently strained by the substrates when ǀΔ*a*/*a*ǀ < 1% (DyScO_3_, GdScO_3_, NdScO_3_), whereas the films partially relaxed when ǀΔ*a*/*a*ǀ > 1% (YAlO_3_, LaAlO_3_, and LSAT). **Figure** **1**a summarizes the out‐of‐plane to the in‐plane lattice parameter ratio (*c*/*a*) for all the films as a function of Δ*a*/*a*. The *c*/*a* ratio largely increases from 0.992 on NdScO_3_ to 1.018 on DyScO_3_ in the coherent (red) region, the *c*/*a*‐change saturates at 1.026 on LSAT, but reduces to 1.025 on LaAlO_3_, and to 1.019 on YAlO_3_ substrates due to lattice relaxation (blue region). We find that *c/a* can be further increased by reducing the thickness *t* of the LTO films when ǀΔ*a*/*a*ǀ > 1%. For example, we varied *t* from 100 nm down to 4 nm on the LaAlO_3_ substrate, and succeeded in further increasing *c/a* from 1.023 up to 1.034 as seen in Figure 1b. The epitaxial growth and atomically flat surface with step‐and‐terrace structures were kept at *t* down to 4 nm (Figures S3 and S4, Supporting Information). We detected Ti^3+^ valence state only in the present LTO films by electron energy loss spectroscopy (EELS) analysis with high‐angle annular dark field scanning transmission electron microscopy (HAADF‐STEM) ( Figure S5a–d, Supporting Information) and confirmed the homogeneity of the La, Ti, and O chemical composition by field‐emission scanning Auger electron spectroscopy (FE‐AES) along the depth direction ( Figure S5e, Supporting Information). These results guarantee the obtained films are of oxygen stoichiometric within the detection sensitivity, but there remains possibility that an unnoticeable amount of defects has an effect on carrier transport properties of the LTO films under epitaxial strain.

For all the films, we estimate the epitaxial strains *ε_xx_* = (*a*_film_ − *a_c_*_/_ *_a_*_=1_)/*a_c_*_/_ *_a_*_=1_ and *ε_zz_* = (*c*_film_ − *c_c_*_/_ *_a_*_=1_)/*c_c_*_/_ *_a_*_=1_ for the in‐plane and the out‐of‐plane directions, respectively, with respect to the fictitious cubic reference with *c/a* = 1 (Figure 1c,d). The in‐plane *ε_xx_* is systematically controlled from +0.36% (tensile strain) to −1.70% (compressive strain) and varies approximately linearly with *c/a*. The out‐of‐plane *ε_zz_* has the opposite sign and reaches up to 1.69% under in‐plane compression. Notably, however, *ε_zz_* becomes virtually constant for LTO films with *t* thinner than 10 nm on LaAlO_3_ substrate (i.e., *c*/*a* > 1.03). These strains translate into Poisson ratios *ν* = *ε_zz_* / (*ε_zz_* − 2*ε_xx_*) **≳** 0.4 for the films with *c*/*a* < 1.03, but that drastically shrinks with *t* to reach *ν* ≈ 0.33 for the thinnest LTO film on LaAlO_3_ substrate (inset to Figure 1e). The consequences for the perovskite unit cell volume *V* are shown in Figure 1e. Consistent with ν < ½, LTO thin films are not volume‐conserving and *V* shrinks with growing *c/a*. Following the sharper decreases in the Poisson ratio at larger *c*/*a*, the volume compression accelerates for thinner films. In this work, we exploit the enhanced compressibility of LTO thin films on LaAlO_3_ substrate to generate larger structural anisotropies *c/a*.

**Figure 2:**
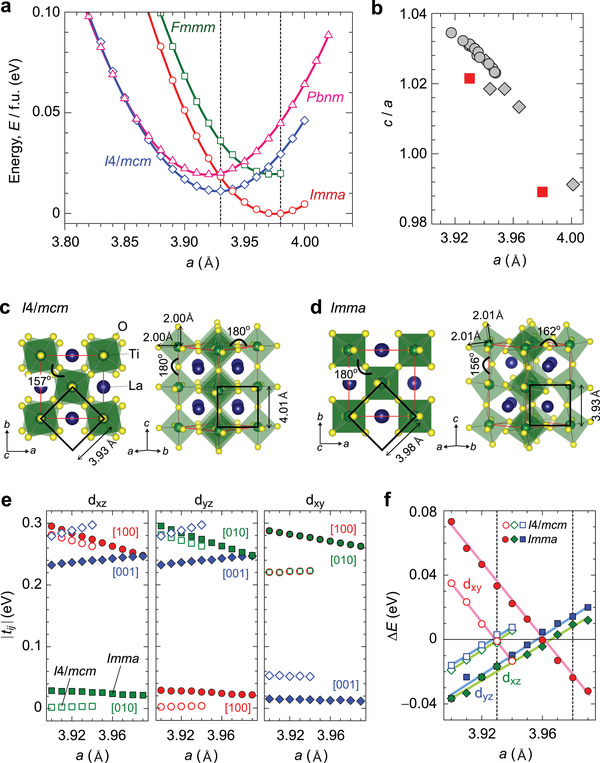
Epitaxial strain dependences of DFT total energy and electronic structure of LTO through varying the in‐plane lattice parameter *a*. a) Calculated total energy (*E*) of LTO for tetragonal *I*4/*mcm* (octahedral rotation *a* ^0^ *a* ^0^ *c* ^−^ in Glazer's notation), and orthorhombic *Imma* (*a* ^0^ *b* ^−^ *b* ^−^), *Fmmm* (*a* ^−^ *b* ^0^ *b* ^0^), *Pnma* (*a* ^+^ *b* ^−^ *b* ^−^) structures. b) Calculated *c*/*a* values at the equilibrium *a* values (the red squares and the *E* minima indicated by the dashed lines in (a)). Experimental data are shown for comparison (the gray circles and diamonds). c,d) Relaxed crystal structures of *I*4/*mcm* c) and *Imma* d). e) Strain dependence of Ti 3d *t*_2g_ nearest‐neighbor hopping parameters, |*t_ij_* |. f) Strain dependence of the on‐site energy difference (crystal‐field) Δ*E* for d*_xy_*, d*_xz_*, and d*_yz_* orbitals with respect to the orbital average energy (*E*_d_ *_xy_* + *E*_d_ *_xz_* + *E*_d_ *_yz_*)/3 for *I*4/*mcm* (open) and *Imma* (closed symbols).

Besides tuning *c/a* and *V*, epitaxial strain may lead to atomic relaxations that lie outside the restrictions of the bulk space group. For a better understanding, we calculated total energetics of LTO for different symmetries from first principles, using density functional theory (DFT) (for details see the Experimental Section). **Figure** **2**a displays the total energy as a function of the in‐plane lattice parameter *a*, where *c* axis length and internal coordinates were relaxed so as to take minimum total energy. We identify two phases that are predicted to be more stable than the bulk structure *Pbnm*, i.e., *I*4/*mcm* with the rotational pattern *a* ^0^ *a* ^0^ *c* ^−^ (in the Glazer notation) for compressive strain and *Imma* with *a* ^0^ *b* ^−^ *b* ^−^ for tensile strain. At equilibrium lattice parameters (the dashed lines at total energy minima for *I4mcm* at *a* = 3.93 Å and for *Imma* at *a* = 3.98 Å in Figure 2a), the calculated *c/a* agrees satisfactory with the experimental data within a 1% error (Figure 2b). The predicted stable structures are depicted in Figures 2c,d. While octahedral distortions are negligible (all Ti–O distances ≈2.0 Å), epitaxial strain causes the Ti–O_6_ octahedra to rotate in the *a*‐*b* (out of a‐b) plane in the *I*4/*mcm* (*Imma*) phase, with the in‐plane and the out‐of‐plane Ti–O–Ti angles of 157° and 156°, respectively.

Next, we discuss how these strain‐induced crystallographic changes modify the electronic structure, focusing on the bandwidth (kinetic energy), the anisotropy (effective dimensionality), and crystal fields (degeneracies). Figure 2e,f shows the Ti 3d‐*t*_2g_ (d*_xy_*, d*_xz_*, d*_yz_*) nearest‐neighbor hopping parameters *t_ij_* and the relative *t*_2g_ crystal fields *ΔE* under strain, obtained from a Wannier projection (Figures S9 and S10, Supporting Information). The in‐ versus out‐of‐plane Ti–O–Ti distortion naturally explains why the d*_xy_* (d*_xz_*, d*_yz_*) hopping parameters are smaller (larger) in the *I*4/*mcm* than in the *Imma* structure. Under compression, the in‐plane (out‐of‐plane) *t_ij_* are largely enhanced (reduced) (Figure 2e), resulting in a small net increase in bandwidth/kinetic energy of 7.5% (1.1%) for the shown range of *Imma* (*I4/mcm*) lattice parameters ( Figure S11, Supporting Information). The anisotropic hopping parameters make the electronic structure more 2D‐like, which we quantify with the measure α^[^ ^28^ ^]^—a ratio of directional hopping parameters giving α = 1 for undistorted 3D‐cubic systems and α = 0 for independent 2D layers. For the *Imma* (*I4/mcm*) lattice shrinking from *a* = 3.99 to 3.90 Å (3.94 to 3.90 Å), α reduces from 3D‐like 0.95 (0.87) down to 0.77 (0.79). The electronic anisotropy of LTO grows with strain, but remains moderate compared to, e.g., the layered iron pnictides, where α ≤ 0.3.^[^ ^28^ ^]^

Finally, strain notably changes the energy levels of the Ti 3d *t*_2g_ (d*_xy_*, d*_xz_*, d*_yz_*) orbitals (Figure 2f). At equilibrium lattice parameter under tensile strain (the dashed line at *a* = 3.98 Å), the d*_xy_* orbital is lowest in energy, as found for bulk LTO.^[^ ^22^ ^]^ Importantly, compressive strain tunes and, eventually, inverts this t_2g_ orbital splitting,^[^ ^23^ ^]^ resulting in a charge‐transfer from d*_xy_* to the (at 300 K quasidegenerate) d*_xz_* and d*_yz_* orbitals. Crystallographic details notwithstanding, the qualitative effect of strain onto hoppings and crystal fields is hence stable irrespective of the realized space‐group under strain, see also Ref. [23].

**Figure 3:**
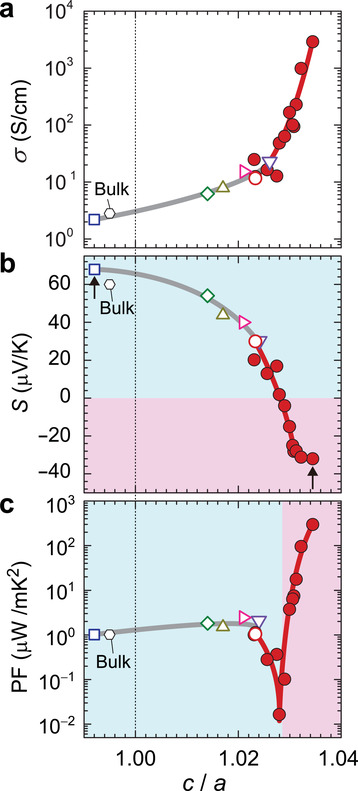
Epitaxial strain dependences of thermoelectric properties of LTO films at room temperature. a–c) *c*/*a* dependence of a) electrical conductivity (*σ*), b) thermopower (*S*), c) power factor (PF) for LTO films with thickness of 50 nm on YAlO_3_ (open pink symbols), LaAlO_3_ (open red symbols), LSAT (open purple symbols), DyScO_3_ (open bright yellow symbols), GdScO_3_ (open green symbols), NdScO_3_ (open blue symbols) and those with thicknesses of 4−100 nm on LaAlO_3_ substrate (closed red circles). *σ*, *S*, and PF for bulk LTO are also shown.^[^ ^29^, ^30^ ^]^ Red (blue) delimits the region of n‐type (p‐type) charge polarity. The vertical arrows in **b** indicate the samples to be discussed in Figure 4.

**Figure** **3**a,b shows the *c*/*a* dependence of a) *σ* and b) *S* at room temperature (RT) for the LTO films on different substrates (open symbols) and those with varying *t* on LaAlO_3_ substrates (closed red circles). With growing *c*/*a*, *σ* drastically increases from 2.2 S cm^−1^ (*c*/*a* = 0.993, tensile strain), which is close to the bulk value 3 S cm^−1^ (the black hexagons)^[^ ^29^ ^]^, up to notable 2920 S cm^−1^ (*c*/*a* = 1.034, compressive strain). The *S* of bulk LTO is positive with *S*_bulk_ = +60 µV K^−1^.^[^ ^30^ ^]^ Also LTO films with small *c/a* have positive *S*, but its magnitude shrinks for increasing *c/a*. At a critical value *c*/*a* ≈ 1.028, *S* eventually changes its sign and reaches −40 µV K^−1^ for the thinnest LTO film on LaAlO_3_. LTO films on YAlO_3_ and LSAT with ǀΔ*a*/*a*ǀ > 1%, qualitatively display the same *S* sign change ( Figure S12, Supporting Information). For substrates with smaller ǀΔ*a*/*a*ǀ < 1%, however, *S* and *c*/*a* are independent of *t*. We thus identify the *c/a* ratio as a direct control parameter of the *S* in strained LTO.

Figure 3c displays the *c*/*a* dependence of PF = *S^2^σ*: With increasing *c*/*a* up to ≈1.02, PF grows moderately because *σ* increases. Beyond the carrier polarity change at *c*/*a* = 1.028, PF is enhanced up to 300 µW m^−^^1^K^−2^, which is >10^2^ times larger than 1 µW m^−^^1^K^−2^ of LTO bulk. This spectacular boost in PF owes to the simultaneous increase of *σ* and *S* in the n‐type region, which defies common wisdom. Usually, the optimization of PF follows a trade‐off relation between *σ* and *S*.^[^ ^10^ ^]^ Indeed, *σ* is larger in metals while *S* is larger in insulators due to the opposing tendency with carrier concentration. This trade‐off relation between *σ* and *S* usually only allows PF optimizations to reach local maxima. Here, in the compressively strained LTO films with *c*/*a* > 1.028 in the n‐type region, the behaviors of *σ* and *S* are apparently decoupled, and PF can be enlarged globally.

**Figure 4:**
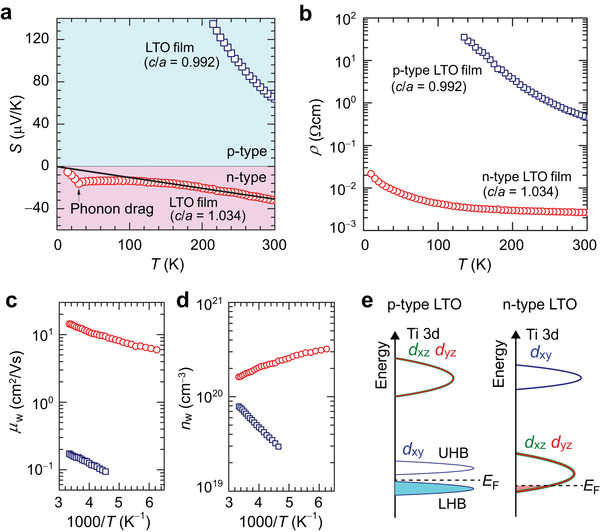
Temperature (*T*) dependences of a) thermopower (*S*) and b‐d) carrier transport properties for 50 nm thick p‐type LTO film (*c*/*a* = 0.992) on NdScO_3_ substrate and 4 nm thick n‐type LTO film (*c*/*a* = 1.034) on LaAlO_3_ substrate. b) *T* dependences of resistivity (*ρ*). c) *T* dependences of weighted mobility (*μ*_w_). d) *T* dependences of carrier concentration (*n*_w_) calculated by *n*_w_ = 1 / (e*ρ μ*_w_). e) Schematic electronic structure of p‐type LTO and n‐type LTO with Ti 3d^1^ electronic configuration.

**Figure** **4**a shows the temperature (*T*) dependences of *S* for the p‐type LTO film (*c*/*a* = 0.992, tensile strain, indicated by the vertical arrow on the left side of Figure 3b) and the n‐type LTO film (*c*/*a* = 1.034, compressive strain, indicated by the vertical arrow on the right side of Figure 3b). The *T* variations of |*S*| show opposite trends, i.e., |*S*| decreases with increasing *T* for the p‐type LTO film, while increases with increasing *T* for the n‐type one. As known from the Boltzmann transport theory based on the carrier‐diffusion model, the electronic contribution of *S* in non‐degenerated semiconductors is expressed as $S\;=\;\frac{k_{B}}{e}\left(\frac{E_{F}-E_{C}}{k_{B}T}+A\right)$, where *k*_B_ is the Boltzmann constant, *e* is the elementary electric charge, *E*_C_ is the conduction band edge energy, and *A* is a transport constant that depends on the dominant scattering mechanism. While, *S* for metals or degenerate semiconductors is basically expressed as $S\;=\;\frac{\pi^{2}k_{B}^{2}T}{3e}{\left\{\frac{\partial [\log(\sigma (E))]}{\partial E}\right\}}_{E_{F}}$ (Mott's equation).^[^ ^31^, ^32^ ^]^ The above results indicate the p‐type LTO film corresponds to the semiconducting *T* dependence, which is consistent with that *E*_F_ locates in the Mott gap for bulk LTO. On the other hand, the n‐type LTO film corresponds to a metallic *T* dependence, suggesting that the Mott gap is closed in the n‐type LTO film. Note that we can find a small *S* peak at *T* = 25 K in the *S*–*T* curve of the n‐type LTO film, which should originate from the phonon‐drag effect.^[^ ^33^ ^]^

Figure 4b–d shows *T* variations of carrier transport properties. Since it was difficult to measure reliable Hall voltages for all the LTO films presumably due to high carrier concentrations (i.e., small Hall voltage) and low carrier mobility, we estimate the weighted mobility (*μ*_w_) using the equation $\mu_{w}=\frac{3h^{3}\sigma }{8\pi {\left(2m_{e}k_{B}T\right)}^{3/2}}\;\left[\frac{\exp[\frac{\left|S\right|}{k_{B}/e}-2]}{1+\exp[-5(\frac{\left|S\right|}{k_{B}/e}-1)]}+\frac{\frac{3}{\pi^{2}}\;\frac{\left|S\right|}{k_{B}/e}}{1+\exp[5(\frac{\left|S\right|}{k_{B}/e}-1)]}\right]$, where *h* is Plank constant and *m*_e_ is the free electron mass.^[^ ^34^ ^]^ The *μ*_w_ is related to the drift mobility *μ* by $\mu_{w}\approx \mu {\left(\frac{m^{*}}{m_{e}}\right)}^{3/2}$, where m* is the density of states effective mass. The corresponding carrier density *n*_w_ is calculated by *n*_w_ = *σ*/(*eμ*_w_). Although the *T* dependences of resistivity (*ρ*) for both the p‐type and the n‐type LTO films show similar semiconducting behaviors as seen in Figure 4b, this is an apparently wrong conclusion. Indeed, although *μ*_w_ shows a similar trend with *T* for the p‐type and the n‐type LTO films (Figure 4c), *n*_w_ shows opposite *T* dependences (Figure 4d). The Arrhenius plot of *n*_w_ (i.e., log *n*_w_–1000/*T* plot) shows a good straight line with the activation energy of 63 meV for the p‐type LTO film, further supporting the conclusion that the p‐type LTO film is a semiconductor with a finite Mott gap. On the other hand, the *T* dependence of *n*_w_ is small for the n‐type one, being consistent with the above conclusion that the n‐type LTO has a metallic electronic structure.

Here, we discuss why the electronic structure of LTO film changes from the Mott insulator state to the metallic state by compressive strain. The mechanism for Mott‐insulating bulk LTO is explained by a combination of small bandwidths and reduced charge fluctuations through a Ti 3d *t*_2g_ orbital splitting, where the d*_xy_* orbital has the lowest energy.^[^ ^22^ ^]^ Our first‐principles calculation results in Figure 2f demonstrated that compressive strain (growing *c/a*) increases bandwidths, while also allowing for more charge fluctuations by doubling the degeneracy of the lowest *t*_2g_ orbital, where the d*_xz_* and d*_yz_* orbitals have the lowest energy. Figure 4e schematically depicts the electronic structures of the p‐type LTO and the n‐type LTO with Ti 3d^1^ electronic configuration based on these results. For the p‐type LTO, the *E*_F_ locates near the midgap energy between the lower and the upper Hubbard bands of the d*_xy_* orbitals, where the *E*_F_ slightly shifts to the lower Hubbard band and leads to the p‐type semiconductor behavior. On the other hand, for the n‐type LTO, it has a metallic electronic structure, where the one d electron occupies a hybridized band of d*_xz_* and d*_yz_* character, and thus it shows n‐type conduction.

**Figure 5:**
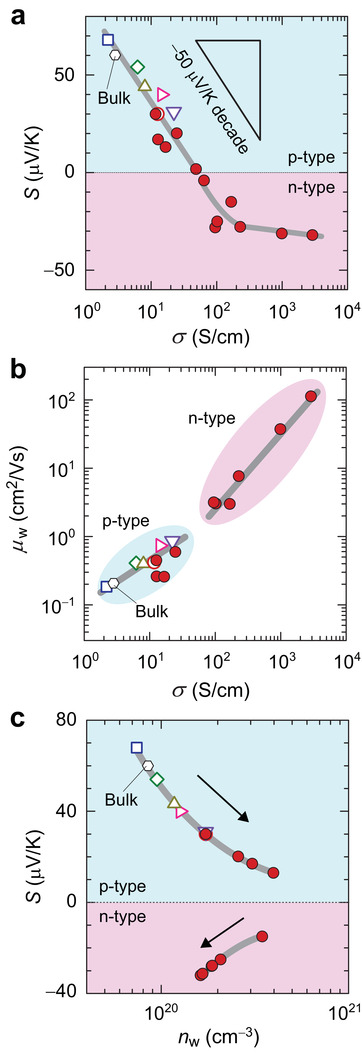
a) Thermopower (*S*) as a function of electrical conductivity (*σ*), b) weighted mobility (*μ*_w_) versus *σ*, and c) *S* versus carrier concentration (*n*_w_) calculated by *n*_w_ = *σ* / (e*μ*_w_) at room temperature for LTO films with thickness of 50 nm on YAlO_3_ (open pink symbols), LaAlO_3_ (open red symbols), LSAT (open purple symbols), DyScO_3_ (open bright yellow symbols), GdScO_3_ (open green symbols), NdScO_3_ (open blue symbols), and those with thicknesses of 4−100 nm on LaAlO_3_ substrate (closed red circles). Red (blue) delimits the region of n‐type (p‐type) charge polarity.

Next, we discuss the origin of the breaking of the trade‐off relation. **Figure** **5**a summarizes the relationship between *S* and *σ* for all the LTO films at RT. In the p‐type region, the *S* linearly decreases as a function of log *σ* with a slope of −50 µV K^−1^ decade^−1^ and passes through zero, resulting in the carrier polarity change from p‐type to n‐type. On the other hand, the unusual simultaneous increase in |*S*| and *σ* is observed in the highly conductive n‐type region, which cannot be explained by the carrier‐diffusion model. However, this relation should be reinterpreted in term of carrier density *n*_w_, similar to Figure 4b–d. As mixed carrier conduction (i.e., electrons and holes contribute to electronic conduction) is expected in the vicinity of the p–n transition, *μ*_*w*_ and *n*_w_ values were calculated with the data apart from the p–n transition. As shown in Figure 5b, *μ*_w_ largely increases with increasing *σ*, and farther larger *μ*_w_ are observed in the n‐type region compared to those in the p‐type region, which is similar to perovskite oxides where *μ*_w_ is larger in n‐type region.^[^ ^35^ ^]^ Then, we can reinterpret the *S–σ* relation in Figure 5a by the *S–n_w_* plots in Figure 5c, which reproduces usual *S–n* relations known as Jonker plot, where |*S*| decreases with increasing *n*_w_, in both the p‐type and the n‐type regions.

Finally, we can conclude that, in the n‐type region, both *S* and *σ* increase simultaneously with increasing *c*/*a* ratio, which breaks the trade‐off relation of the carrier‐diffusion model, because *S* is increased by the reduced *n*_w_ as described in the carrier‐diffusion model, while *σ* is also increased by the enhancement of *μ*_w_ that surpasses the reduction in *n*_w_. Note that the simultaneous increase in *S* and *σ* is previously reported in (Sr_0.85_La_0.15_)TiO_3−_ *_x_* films, where the oxygen vacancy formation increases *σ*, while *S* is also increased due to the polaron mass enhancement.^[^ ^36^ ^]^

## 3 Conclusion

In summary, we studied the thermoelectric response of LTO thin films by varying their electronic structure from a Mott insulator to a metallic state through selection of substrate lattice mismatches and reducing film thicknesses, and found the simultaneous increase in |S| and *σ* is realized by large enhancement of carrier mobility beyond the Mott insulator to metal transition, boosting the power factor by more than 2 orders of magnitude. Our experiments suggest that epitaxial strain will be an ideal tool to finely tune materials close to their Mott transition, and thus to harvest large power factors from TMOs that are inconspicuous in their bulk.

## 4 Experimental Section

### Thin Film Growth

LTO epitaxial films were grown on (001) pseudo‐cubic perovskite substrates of YAlO_3_, LaAlO_3_, LSAT, DyScO_3_, GdScO_3_, and NdScO_3_ (10 × 10 × 0.5 mm^3^) by pulsed laser deposition. A KrF excimer laser (*λ* = 248 nm) was used to ablate a La_2_Ti_2_O_7_ polycrystalline target disk, with a laser energy fluence and repetition rate of 1 J cm^−2^ and 2 Hz, respectively. Films were deposited in vacuum (≈1 × 10^−5^ Pa) at a growth temperature of 780 °C. After deposition, films were cooled to RT in vacuum.

### Structural and Chemical Analysis

The crystal structures were investigated by high‐resolution XRD (anode radiation: monochromatic CuKα_1_) at RT (Figures S1–S3, Supporting Information). Atomic force microscopy (AFM) revealed atomically flat surfaces with step‐and‐terrace structures ( Figure S4, Supporting Information). TEM samples with 50 nm thick LTO films on LaAlO_3_ substrate were prepared by mechanical polishing with cooling water and thinned by Cryo. ion slicer with a holding temperature of −150°C at ≈10^−3^ Pa. The cross‐sectional microstructure of the LTO film was examined at RT by HAADF‐STEM (JEM‐ARM200F, 200 kV, JEOL), with electron incident direction LaAlO_3_ [100]. Dominance of the Ti^3+^ valence state was confirmed by EELS in conjunction with HAADF‐STEM that also attested the sharpness of the LTO/LaAlO_3_ interface (Figures S5–S7, Supporting Information). HAADF‐STEM images were taken with the detection angle of 68−280 mrad, and the EELS were acquired by Enfinium spectrometer (Gatan Inc.) with the energy resolution of about 1 eV. Homogeneity of the La, Ti, and O chemical compositions was verified by FE‐AES along the depth direction ( Figure S5e, Supporting Information).

### Electrical Transport Measurement

*σ* was measured by a d.c. four‐probe method with the van der Pauw electrode configuration. *S* was measured by applying temperature gradient (Δ*T*) of ≈4 K while the actual temperatures of both sides of the film surface were monitored by thermocouples. The thermo‐electromotive force (Δ*V*) and Δ*T* were simultaneously measured, and *S* was obtained from the slope of the Δ*V*−Δ*T* plots.

### First‐Principles Calculation

The stable structure of LTO under epitaxial strain and their electronic structures were examined by first‐principles calculations. All structural relaxations are performed within the generalized gradient approximation (GGA, in the Perdew–Burke–Ernzerhof (PBE) realization) of DFT using the QUANTUM ESPRESSO code,^[^ ^37^ ^]^ which implements the plane‐wave pseudo‐potential method. We employed pseudo‐potentials from the SSSP library^[^ ^38^ ^]^ with an energy cutoff of 50 Ry. Structural relaxations of LTO under different epitaxial constraints were simulated using a conventional cell containing 20 atoms with a Γ‐centered 9 × 9 × 6 *k*‐point mesh. For each strain, we fixed the in‐plane lattice parameter and relaxed the out‐of‐plane lattice parameter *c* as well as all internal positions. The results were double checked with the GGA‐PBEsol functional ( Figure S8, Supporting Information). Strain dependencies of nearest‐neighbor hopping parameters and on‐site energies of the Ti 3d *t*_2g_ orbitals were evaluated from maximally localized Wannier functions from Wannier90,^[^ ^39^ ^]^ using Kohn‐Sham states within −1 eV – +6 eV around the *E*_F_, and frozen windows within −1 to −0.35 eV and −1 to +0.1 eV for *I4/mcm* and *Imma* structures, respectively.

## Conflict of Interest

The authors declare no conflict of interest.

## Supporting information



Supporting Information

## Data Availability

Research data are not shared.
