# Supplementary material for: Breaking of Thermopower–Conductivity Trade‐Off in LaTiO3 Film around Mott Insulator to Metal Transition
Source: Adv Sci (Weinh). 2021 Oct 21;8(23):2102097. doi: 10.1002/advs.202102097 (PMC8655177; doi:10.1002/advs.202102097)
Supplement: Supplementary file 1 — Supporting Information [file ADVS-8-2102097-s001.pdf]

ADVANCED SCIENCE
Open Access

Supporting Information

for *Adv. Sci.*, DOI: 10.1002/advs.202102097

Breaking of thermopower–conductivity trade-off in $LaTiO_3$ film around Mott insulator to metal transition

Takayoshi Katase, Xinyi He, Terumasa Tadano, Jan M. Tomczak, Takaki Onozato, Keisuke Ide, Bin Feng, Tetsuya Tohei, Hidenori Hiramatsu, Hiromichi Ohta, Yuichi Ikuhara, Hideo Hosono, and Toshio Kamiya**

Copyright WILEY-VCH Verlag GmbH & Co. KGaA, 69469 Weinheim, Germany, 2018.

Supporting Information

Supporting Figures S1–S12

Breaking of thermopower – conductivity trade-off in $LaTiO_3$ film around Mott insulator to metal transition

Takayoshi Katase, Xinyi He, Terumasa Tadano, Jan M. Tomczak, Takaki Onozato, Keisuke Ide, Bin Feng, Tetsuya Tohei, Hidenori Hiramatsu, Hiromichi Ohta, Yuichi Ikuhara, Hideo Hosono, and Toshio Kamiya**

Supporting Information

Substrate selection

Note that the generation of a metallic state has been observed in heterostructures of LTO ultrathin layers (1–4 u.c.) embedded in $SrTiO_3$, which has been explained by charge transfer between Ti^{3+} and Ti^{4+} cations at the interfaces between the two components.[1] In addition, a metallic conductivity has also been seen in 15-45 nm-thick LTO films on $SrTiO_3$, where the sheet carrier density was found to scale linearly with the thickness, suggesting that the metallicity is not just restricted to the interface region.[2,3] However, it has been difficult to assign its origin purely to epitaxial strain-induced electronic structure modification because of the charge discontinuity ($[TiO_2^{-}/LaO^{+}]$ / $[TiO_2^{0}/SrO^{0}]$) at the $LaTiO_3/SrTiO_3$ interface and/or oxygen vacancy formation.[4,5] Therefore, we used insulating substrates that have no charge discontinuity, e.g., ($[TiO_2^{-}/LaO^{+}]$ / $[AlO_2^{-}/LaO^{+}]$) at the $LaTiO_3/LaAlO_3$ interface, and $LaAlO_3$ has furthermore been shown to be stable against reduction even when heated to high temperatures in vacuum.

Stoichiometry, valence, atomic-scale characterization

Excess oxygen and/or cation off-stoichiometry are known as the most critical parameters for the electronic properties of $La_{1-x}TiO_{3+\delta}$.[6-9] The oxygen content in the perovskite $LaTiO_{3+\delta}$ can be varied from 3.0 to 3.5, which changes the formal Ti valence state from 3+ to 4+ and finally produces the $La_2Ti_2O_7$ pyrochlore phase.[7] We then performed high-angle annular dark field scanning transmission electron microscopy (HAADF-STEM) observations and electron energy loss spectroscopy (EELS) analysis of the 50 nm thick LTO film on an $LaAlO_3$ substrate. **Figure S5(a)** shows the cross-sectional HAADF-STEM image. The detailed interface structure is summarized in **Fig. S7**. Due to the large $\Delta a/a$ of –4.95%, the misfit dislocations are seen in the interval of 8 nm at the interface region, where the pseudo-semi

coherent interface is formed at the hetero-interface of LTO and $LaAlO_3$.[10] When it is compared to the time dependence of RHEED oscillation during thin film growth, the structure relaxation occurs at a distance of 2–3 unit cells from the film/substrate interface. The clear atomic structure of the perovskite lattice is seen from near the interface to bulk region after the structure relaxation (**Fig. S5(a)**). The La-La interatomic distances taken from **Fig. S5(a)** along the out-of-plane direction is plotted in **Fig. S6**. The lattice space is homogeneous when the film is relaxed and t exceeds 9 u.c. (~3.6 nm). As the minimum thickness 4 nm already exceeds, and therefore the lattice spacing can be regarded as uniform in the films examined in this study.

Figures S5(b) and **S5(d)** show Ti $L_{2,3}$ edge EELS spectra taken in the bulk region and interface in the HAADF-STEM image. The spectrum shows the two peaks of L_2 and L_3 at 457 eV and 463 eV, respectively. It has been reported that the Ti $L_{2,3}$ EELS spectra of $SrTiO_3$ with Ti^{4+} have four peaks at 457 eV, 459 eV, 462 eV, and 465 eV, while those of LTO with Ti^{3+} have two peaks at 457 eV and 463 eV.[11] The distinctly different Ti $L_{2,3}$ EELS spectra for the Ti^{3+} and Ti^{4+} formal valence states support that only the Ti^{3+} valence state is detected for the present LTO film. In addition, chemical compositional analysis of the film was performed by field-emission scanning Auger electron spectroscopy (FE-AES) along the depth direction (**Fig. S5(e)**). The atomic concentration dependence of La, Ti, Al, and O elements indicates that the elemental distributions in the bulk region from surface is homogeneous and the La/Ti ratio is constant at the resolution level of AES measurements. The atomic concentration of Ti and Al sharply decreases and increases at the film/substrate interface, respectively. These results further support that the increase of σ and the carrier polarity change from p-type to n-type originate from the epitaxial strain induced lattice deformation of LTO film.

Supporting Figures

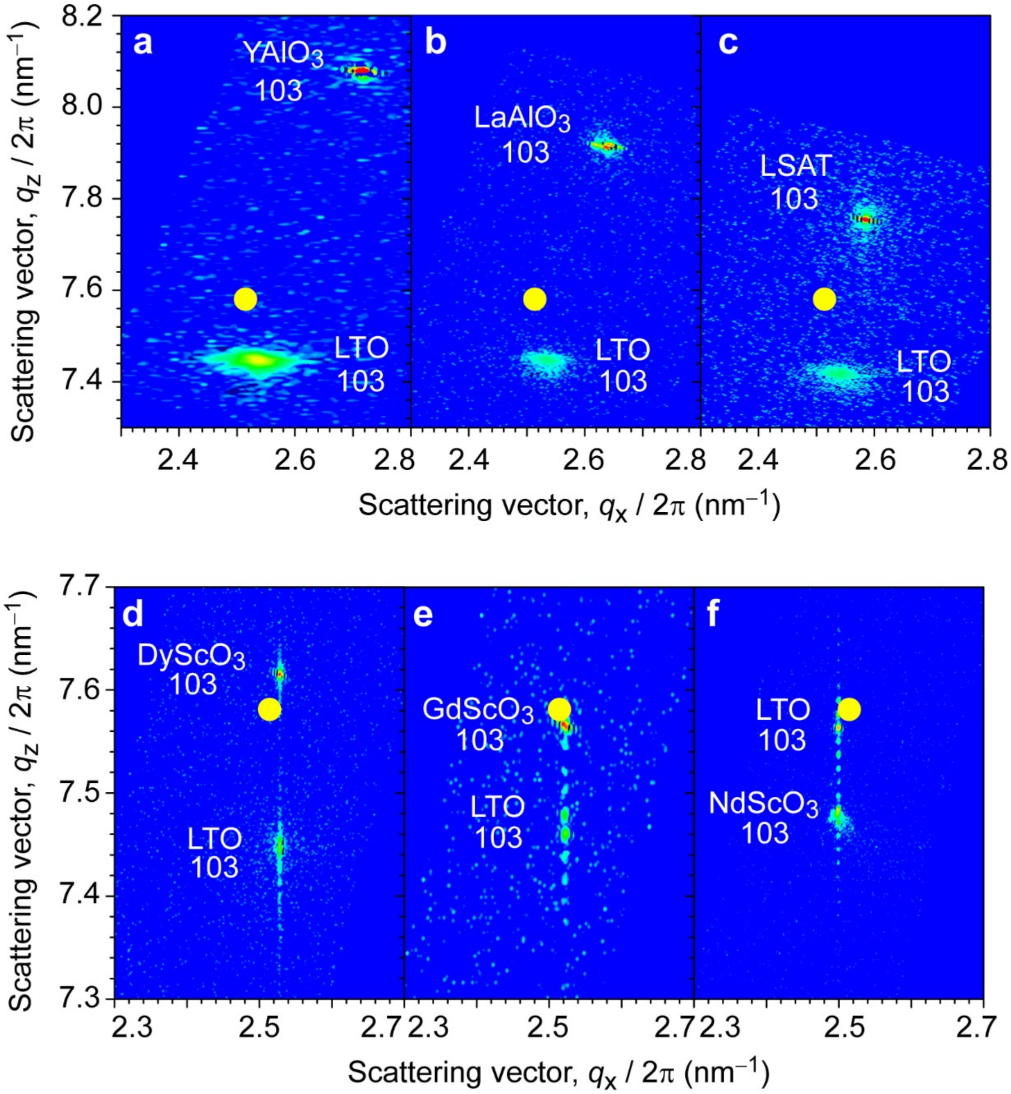

Figure S1. Reciprocal space maps around the 103 reflection in the pseudo-perovskite unit cell for 50 nm thick LTO films grown on (a) $YAlO_3$ ($\Delta a/a = -6.50\%$), (b) $LaAlO_3$ ($\Delta a/a = -4.95\%$), (c) LSAT ($\Delta a/a = -2.44\%$), (d) $DyScO_3$ ($\Delta a/a = -0.93\%$), (e) $GdScO_3$ ($\Delta a/a = -0.43\%$), (f) $NdScO_3$ ($\Delta a/a = +0.58\%$) substrates. The yellow circles indicate the diffraction spots of bulk LTO.[12]

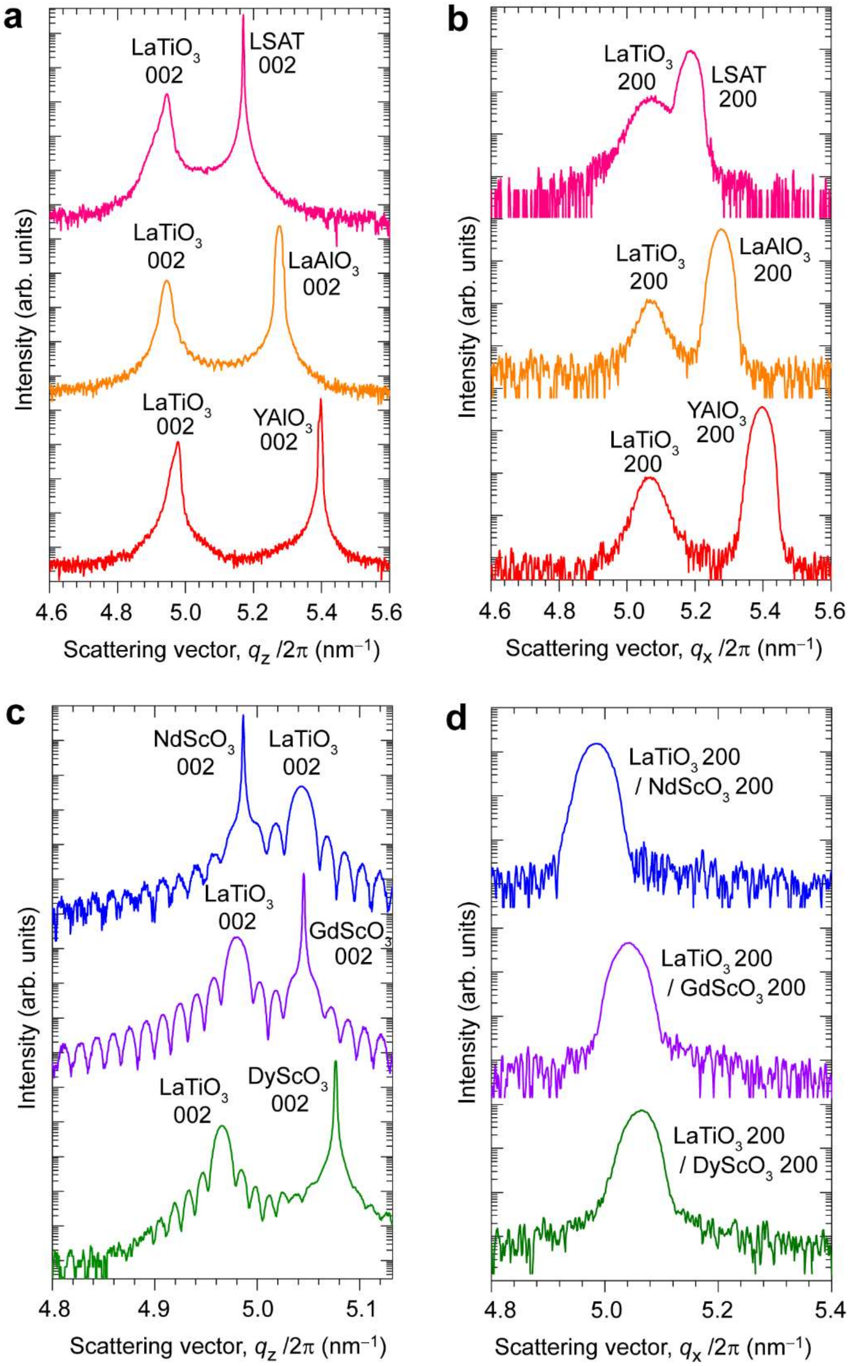

Figure S2. (a,c) Out-of-plane and (b,d) in-plane XRD patterns of LTO films on (a,b) $YAlO_3$, $LaAlO_3$, LSAT substrates with $|\Delta a/a| > 1\%$ and (c,d) $DyScO_3$, $GdScO_3$, $NdScO_3$ substrates with $|\Delta a/a| < 1\%$.

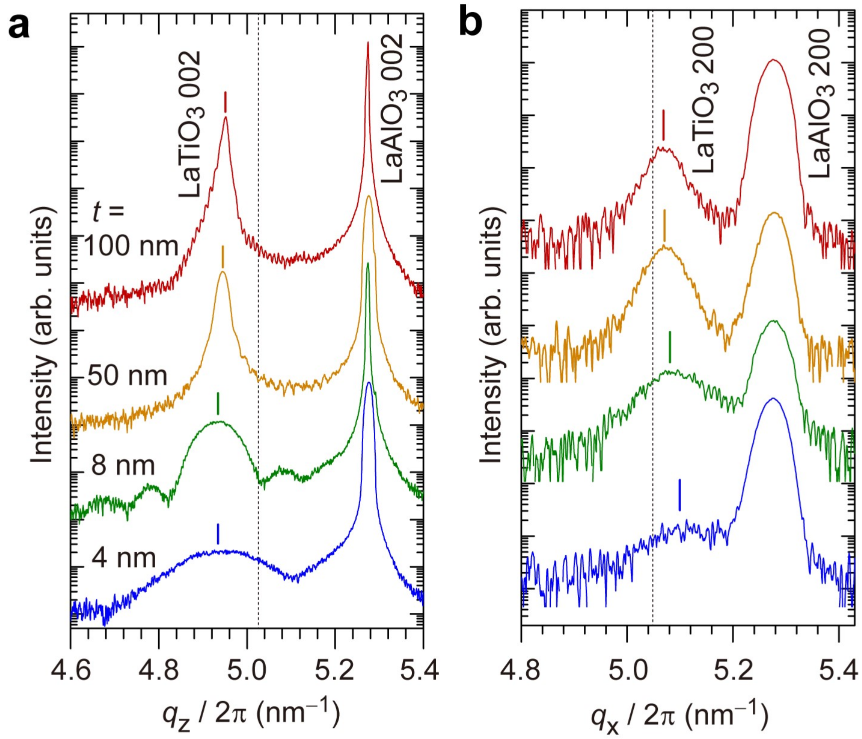

Figure S3. Out-of-plane (a) and in-plane (b) XRD patterns of LTO films with different thicknesses (*t*) of 4 nm, 8 nm, 50 nm, and 100 nm grown on $LaAlO_3$ substrates. The vertical bars around $q_{z,x}$ = 5 nm^{-1} indicate the diffraction peak positions of LTO 002 (a) and LTO 200 (b). The dotted lines indicate the 002 and 200 peak positions of LTO bulk.[12] The positions of 002 and 200 diffraction peaks of LTO films shift to higher and lower scattering vectors, respectively, revealing an increase and a decrease in the lattice spacing of (002) and (200) planes with an increase of *t*, respectively. All the films have the 002 LTO diffraction peak with Kiessig fringes, confirming that the *c*-axis of the film is highly oriented along 002 $LaAlO_3$ and homogeneous for any thickness. For a 50 nm thick film, the full widths at half maximum values of the 002 LTO rocking curve are 0.07°, which confirms the high crystallinity of the films. The ϕ scan of the 200 LTO diffraction displays four peaks periodically appears at every 90° (data not shown), indicating that the LTO film has four-fold rotational symmetry in the *ab*-plane and is epitaxially grown on $LaAlO_3$, consistent with the RHEED patterns (**Fig. S4**).

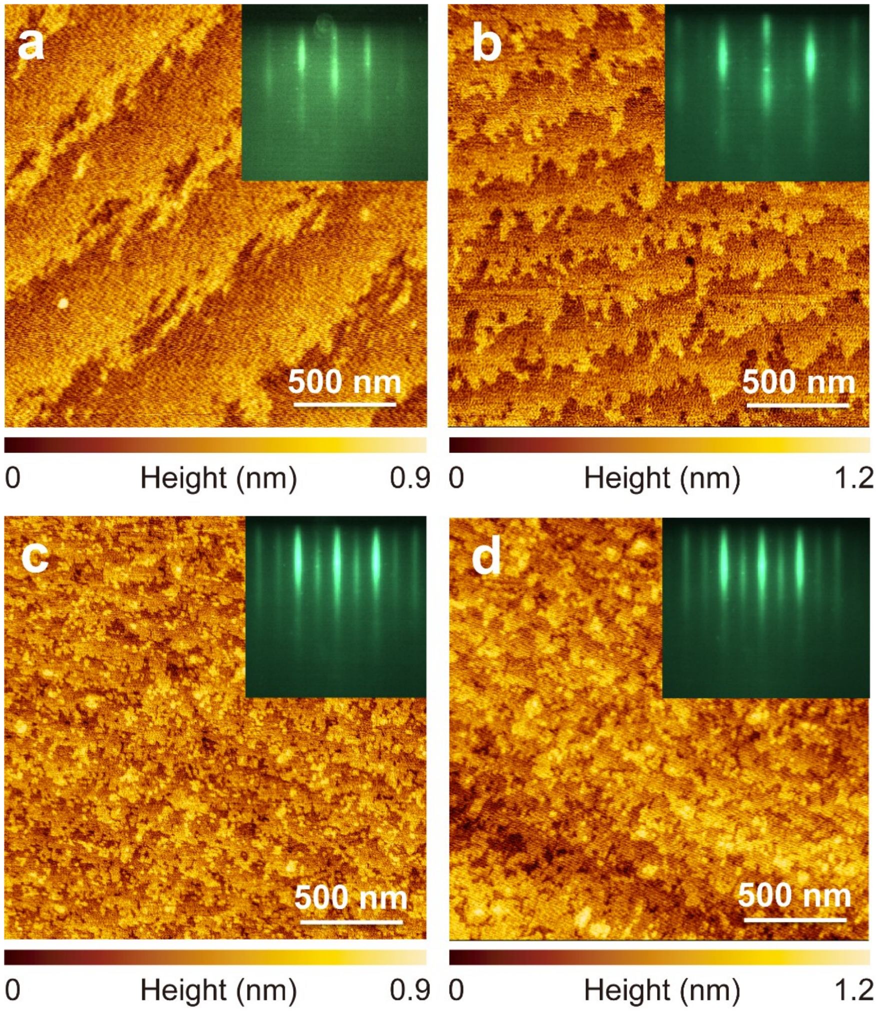

Figure S4. Atomic force microscopy (AFM) images of LTO films with thicknesses of (a) 4 nm, (b) 8 nm, (c) 50 nm, and (d) 100 nm, grown on $LaAlO_3$ substrates. The insets show reflection high-energy electron diffraction (RHEED) patterns taken after the film deposition.

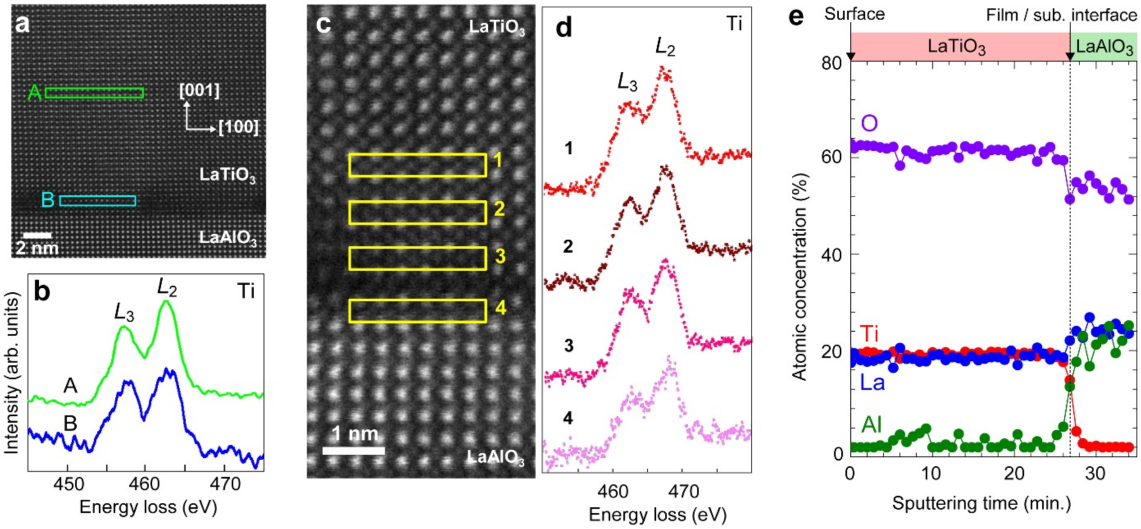

Figure S5. Atomic-scale crystal structure, electronic state, and chemical composition analysis of 50 nm thick LTO film on $LaAlO_3$ substrate. (a) HAADF-STEM image of LTO film. (b) Ti $L_{2,3}$ edge EELS spectra taken at region A and B in (a). (c) Enlarged HAADF-STEM image in the vicinity of the film / substrate interface in (a). (d) Ti $L_{2,3}$ edge EELS spectra taken at regions 1–4 in (c). (e) Depth profiles of atomic concentrations of La, Ti, Al, and O elements measured by AES measured through Ar sputtering as a function of sputtering time. The positions of the film surface and film/substrate interface are indicated at the top of the panel. The incident electron beam was weakly accelerated at 3 kV and 10 nA to suppress an electrical charge-up of the specimens.

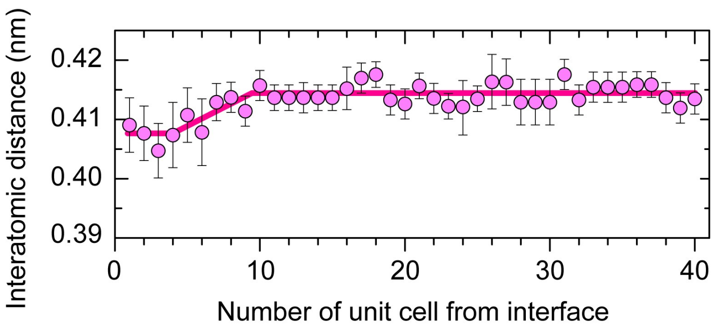

Figure S6. La-La interatomic distance taken from Fig. S5(a) along out-of-plane direction of 50 nm thick LTO film on $LaAlO_3$ substrate. The circles indicate the La-La interatomic distances averaged over 5 layers of perovskite unit cells. The error bars indicate the standard deviations.

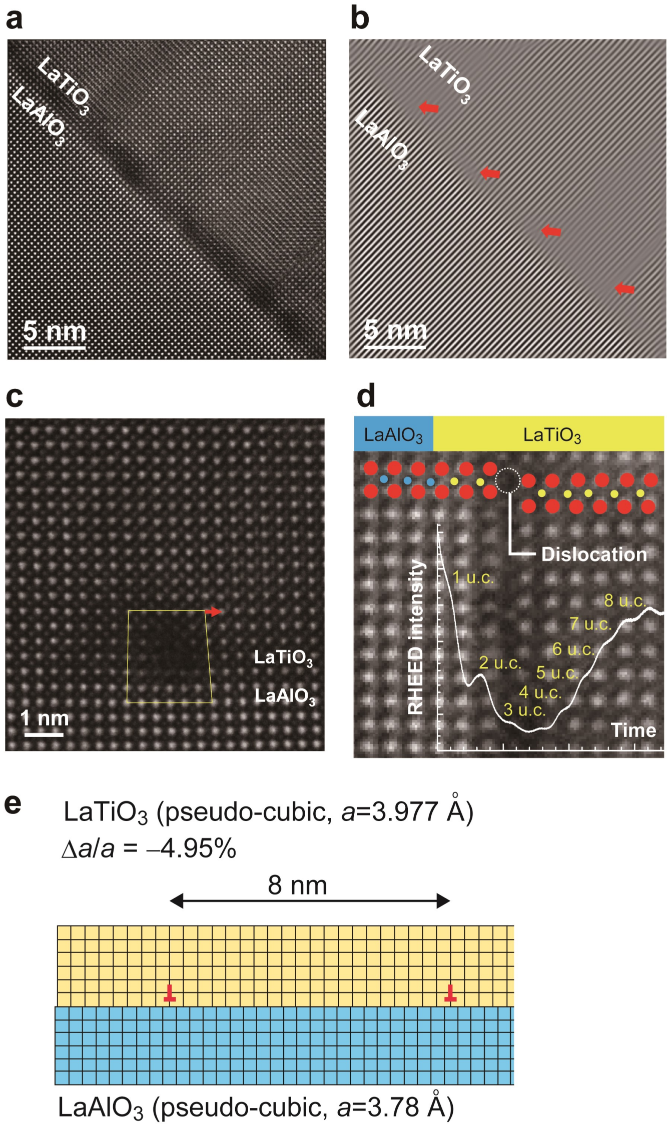

Figure S7. (a) Cross-sectional HAADF-STEM image and (b) Fourier transformation image of (a) for LTO film on $LaAlO_3$ substrate. The arrows in (b) indicate the positions of misfit dislocations. (c,d) Enlarged HAADF-STEM image around the dislocation core (c) and at the film / substrate interface (d). The red arrow in (c) indicates the Burgers vector. (d) The time dependence of RHEED intensity is shown for comparison. (e) Interface structure model for LTO and $LaAlO_3$, where the mismatch dislocation distance is 8 nm.

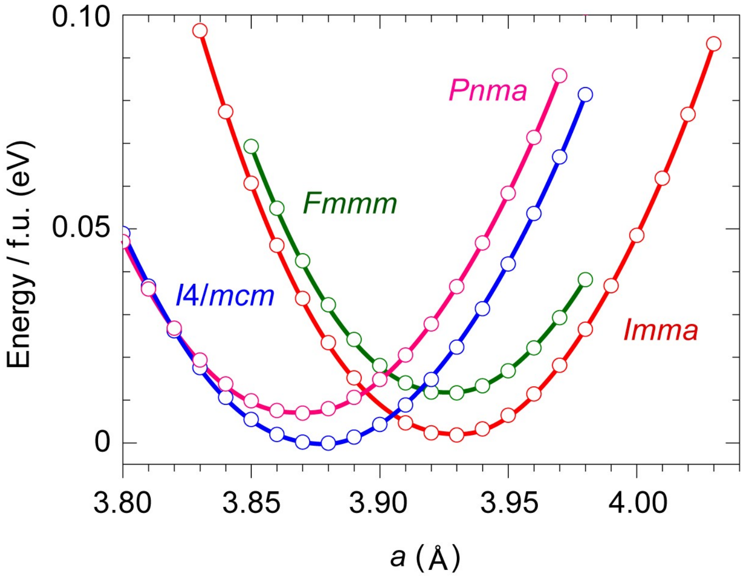

Figure S8. DFT total energies, calculated by GGA-PBEsol, of LTO for tetragonal *I*4/*mcm* phase (octahedral rotation a^0 a^0 c^-), orthorhombic *Imma* phase (a^0 b^- b^-), orthorhombic *Fmmm* phase (a^- b^0 b^0), orthorhombic *Pnma* phase (a^+ b^- b^-) with Glazer's tilt number.

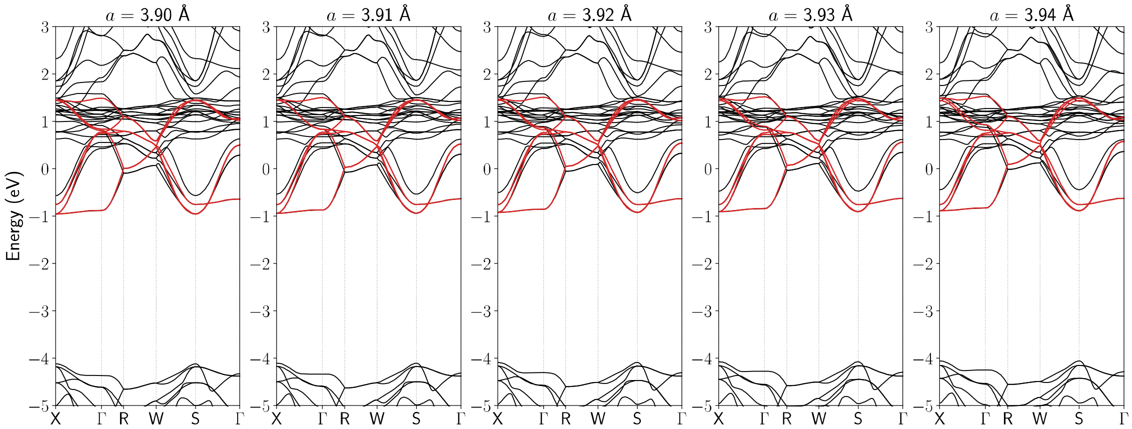

Figure S9. Strain dependence of the band structure for LTO with *I*4/*mcm* structure as a function of *a*. Red lines indicate the Wannier projection onto the Ti 3d t_{2g} states.

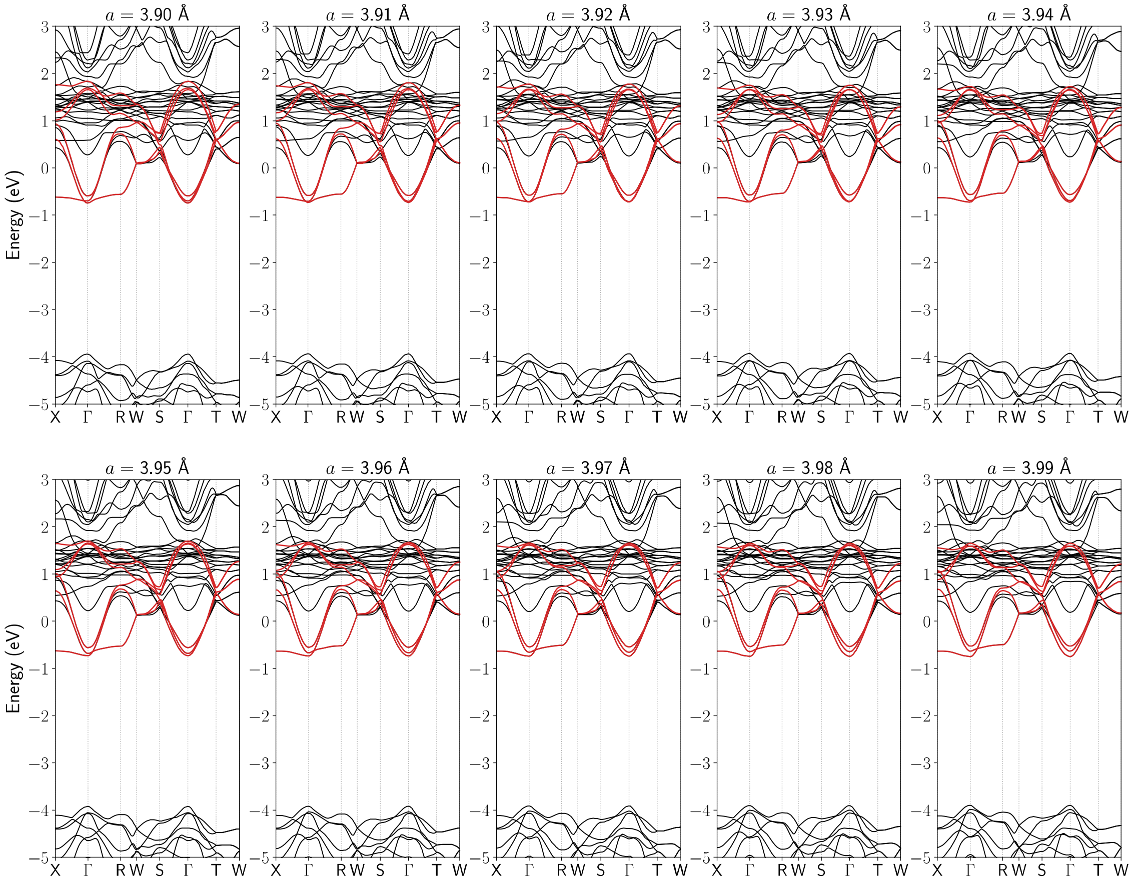

Figure S10. Strain dependence of the band structure for LTO with *Imma* structure as a function of *a*. Red lines indicate the Wannier projection onto the Ti 3d t_{2g} states.

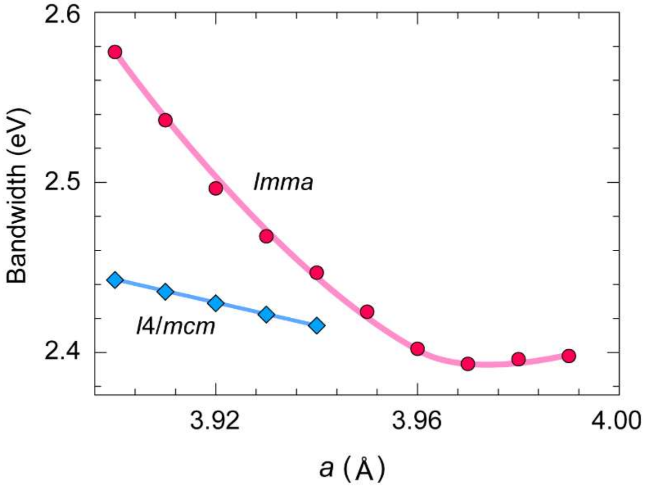

Figure S11. Bandwidth estimated from minimum and maximum values of Ti 3d t_{2g} Wannier eigenvalues in the Γ−X line for *I*4/*mcm* phase (**Fig. S9**) and *Imma* phase (**Fig. S10**).

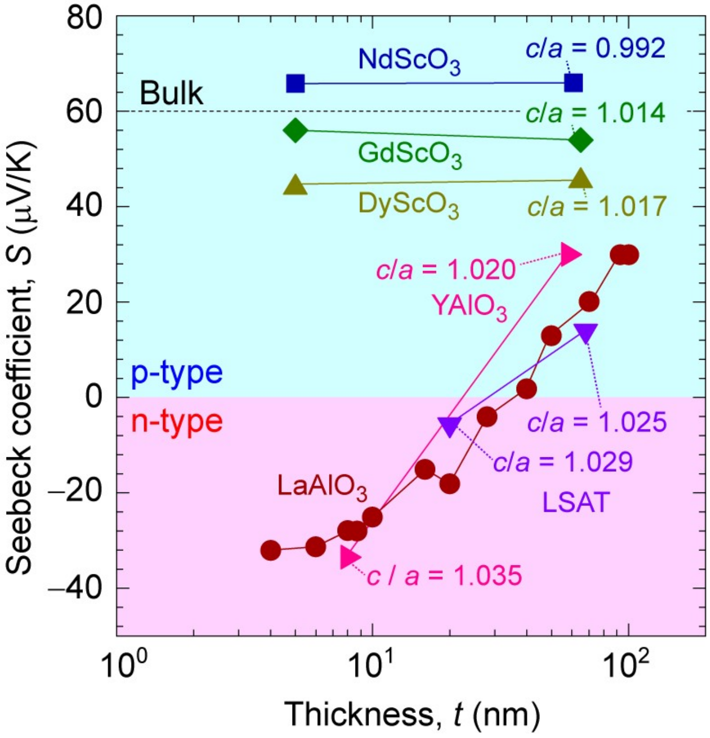

Figure S12. Film thickness (*t*) dependence of thermopower (*S*) for LTO film on $YAlO_3$ (pink symbols), $LaAlO_3$ (dark red symbols), LSAT (purple symbols), $DyScO_3$ (bright yellow symbols), $GdScO_3$ (green symbols), and $NdScO_3$ (blue symbols) at room temperature. The dotted line indicates the $S = +60 \mu V/K$ of LTO bulk.[13]

References

[1] A. Ohtomo, D. A. Muller, J. L. Grazul, H. Y. Hwang *Nature* **2002**, *419*, 378.

[2] F. J. Wong, S.-H. Baek, R. V. Chopdenkar, V. V. Mehta, H.-W. Jang, C.-B. Eom, Y. Suzuki *Phys. Rev. B* **2010**, *81*, 161101.

[3] C. He, T. D. Sanders, M. T. Gray, F. J. Wong, V. V. Mehta, Y. Suzuki *Phys. Rev. B* **2012**, *86*, 081401.

[4] R. Ohtsuka, M. Matvejeff, K. Nishio, R. Takahashi, M. Lippmaa *Appl. Phys. Lett.* **2010**, *96*, 192111.

[5] J. Biscaras, N. Bergeal, S. Hurand, C. Grossetête, A. Rastogi, R. C. Budhani, D. LeBoeuf, C. Proust, J. Lesueur *Phys. Rev. Lett.* **2012**, *108*, 247004.

[6] F. Lichtenberg, D. Widmer, J. G. Bednorz, T. Williams, A. Reller *Z. Phys. B Condens. Mater.* **1991**, *82*, 211.

[7] A. Ohtomo, D. A. Muller, J. L. Grazul, H. Y. Hwang *Appl. Phys. Lett.* **2002**, *80*, 3922.

[8] F. B. Wang, J. Li, P. Wang, X. H. Zhu, M. J. Zhang, Z. H. Peng, S. L. Li, L. P. Yong, Y. F. Chen, X. S. Sun, D. N. Zheng *J. Phys.: Condens. Mater.* **2006**, *18*, 5835.

[9] D. A. Crandles, T. Timusk, J. D. Garrett, J. E. Greedan *Phys. Rev. B* **1994**, *49*, 16207.

[10] Y. Ikuhara, P. Pirouz *Microsc. Res. Tech.* **1998**, *40*, 206.

[11] D. A. Muller, N. Nakagawa, A. Ohtomo, J. L. Grazul, H. Y. Hwang *Nature* **2004**, *430*, 657.

[12] M. Cwik, T. Lorenz, J. Baier, R. Müller, G. André, F. Bourée, F. Lichtenberg, A. Freimuth, R. Schmitz, E. Müller-Hartmann, M. Braden *Phys. Rev. B* **2003**, *68*, 060401.

[13] C. C. Hays, J.-S. Zhou, J. T. Markert, J. B. Goodenough *Phys. Rev. B* **1999**, *60*, 10367.
